# Abdominal functional electrical stimulation to enhance mechanical insufflation-exsufflation

**DOI:** 10.1080/10790268.2015.1114226

**Published:** 2016-11

**Authors:** Euan J. McCaughey, Alan N. McLean, David B. Allan, Henrik Gollee

**Affiliations:** 1 Centre for Health Systems and Safety Research, Australian Institute for Health Innovation, Macquarie University, Sydney, Australia; 2 Centre for Rehabilitation Engineering, School of Engineering, University of Glasgow, Glasgow, Scotland, UK; 3 Scottish Centre for Innovation in Spinal Cord Injury, www.scisci.org.uk, Scotland, UK; 4 Queen Elizabeth National Spinal Injuries Unit, Southern General Hospital, Glasgow, Scotland, UK

**Keywords:** Abdominal muscles, Functional electrical stimulation, Mechanical insufflation-exsufflation, Spinal cord injury, Tetraplegia

## Abstract

**Context:**

Respiratory complications, attributed to the build-up of secretions in the airway, are a leading cause of rehospitalisation for the tetraplegic population. Previously, we observed that the application of Abdominal Functional Electrical Stimulation (AFES) improved cough function and increased demand for secretion removal, suggesting AFES may aid secretion clearance. Clinically, secretion clearance is commonly achieved by using Mechanical insufflation-exsufflation (MI-E) to simulate a cough. In this study the feasibility of combining AFES with MI-E is evaluated.

**Findings:**

AFES was successfully combined with MI-E at eight fortnightly assessment sessions conducted with one sub-acute participant with tetraplegia. By using the signal from a pressure sensor, integrated with the MI-E device, AFES was correctly applied in synchrony with MI-E with an accuracy of 96.7%. Acute increases in exhaled volume and peak flow were observed during AFES assisted MI-E, compared to MI-E alone, at six of eight assessment sessions.

**Conclusion:**

The successful integration of AFES with MI-E at eight assessment sessions demonstrates the feasibility of this technique. The acute increases in respiratory function observed at the majority of assessment sessions generate the hypothesis that AFES assisted MI-E may be more effective for secretion clearance than MI-E alone.

## Introduction

An injury to the cervical region of the spinal cord can cause paralysis affecting all four limbs, termed tetraplegia. Tetraplegia results in impairment or paralysis of the major respiratory muscles, leading to an inability to generate an effective cough. Respiratory complications, attributed to the build-up of secretions in the lungs and airways due to this inability to generate an effective cough, are a leading cause of rehospitalisation for the tetraplegic population.^1^

Inability to generate an effective cough leads to mucus becoming thick and difficult to remove. Clinically, this problem is addressed using either a pharmacological or physical approach. Pharmacologically, antibiotics, mucolytics (medicines that make mucus less sticky and easier to cough up) and the application of drugs via a nebuliser are used to break up secretions. Physically, manually assisted coughing,^2^ mechanical insufflation-exsufflation (MI-E) or, when a tracheostomy is present, tracheal suctioning,^3^ are used to aid secretion break up and removal. MI-E is the application of alternating positive and negative pressure to the user's airway to simulate a cough and break up secretions, with suction applied after each simulated cough to remove these secretions. MI-E has been shown to lead to more effective secretion clearance and a reduction in respiratory complications compared to both manually assisted coughing and tracheal suctioning.^3,4^ Unlike tracheal suctioning, MI-E is capable of removing secretions from both bronchi and has increased comfort for users.^3,5^ MI-E has also been shown to reduce both intensive care length of stay and reintubation rates after mechanical ventilation.^6^ Combining MI-E with another technique that improves respiratory function may enhance the effectiveness of this intervention.

Functional Electrical Stimulation (FES) is the application of a train of electrical pulses to a motor nerve, causing the associated muscle to contract.^7^ The application of FES to the abdominal muscles (Abdominal FES (AFES)), has been shown to achieve an acute increase in cough function.^8,9^ During a previous study it was observed that AFES led to an increased demand for secretion removal,^10^ indicating that AFES was breaking up lung secretions. It was hypothesized that combining AFES with MI-E would enhance the effectiveness of this technique. While systems have been developed that automatically apply AFES in synchrony with the user's respiratory activity,^8,11^ no system has been developed to enable AFES to be automatically applied in synchrony with MI-E. Such a system would make the combination of AFES and MI-E considerably more practical for a clinical setting.

The aim of this single participant case study was to evaluate the feasibility of combining AFES with MI-E.

## Methods

One participant with tetraplegia (male, 69 years old, injury level C6, American Spinal Injury Association Impairment Scale Level A, 26 days post injury), who was an inpatient in a dedicated spinal injuries unit within a university teaching hospital, was recruited for this study. Ethical approval was granted by the local ethics committee, all procedures conformed to the Declaration of Helsinki and the participant provided written informed consent.

### Study protocol

The participant attended eight biweekly assessment sessions, with a total study duration of 14 weeks. At the beginning of each assessment session AFES assisted (stimulated) and unassisted (unstimulated) Forced Vital Capacity (FVC) and Peak Expiratory Flow (PEF) were measured. The participant was instructed to inhale to maximum lung capacity and exhale as fully as possible, with verbal encouragement provided. This manoeuvre was performed five times for both stimulated and unstimulated breaths. Unstimulated FVC was recorded as the largest exhaled volume within 0.15 L of another attempt, while stimulated PEF was the largest exhaled flow rate within 0.67 L/s of another attempt (as recommended by the American Thoracic Society (ATS)/European Respiratory Society (ERS) standards for spirometry).^12^ The same criteria were applied for stimulated FVC and PEF.

MI-E was applied with an insufflation pressure of 20 cmH_2_ *O* for two seconds and an exsufflation pressure of −20 cmH_2_ *O* for three seconds, with a pause of two seconds between each cycle. During each insufflation period the participant was asked to inhale as fully as possible and during each exsufflation period they were asked to exhale as fully and as forcibly as possible. The parameters chosen are based on clinical judgement, with pressures lower than those recommended for standard clinical practice.^3,6^ However, as the participant did not require acute secretion clearance these pressures were chosen to avoid discomfort while still demonstrating the feasibility of the technique.

Following standard clinical protocol, five insufflation-exsufflation cycles were applied during both unassisted and AFES assisted MI-E, to form one run. A second run of MI-E was then performed after a rest period of approximately two minutes, providing a total of 10 stimulated and 10 unstimulated attempts. The volume and flow rate measured during each of the 10 unstimulated attempts were calculated. Using the ATS/ERS standards for spirometry for guidance,^12^ assisted Exhaled Volume (aEV) was denoted as the largest exhaled volume from these 10 attempts which was within 0.15 L of another attempt. Assisted Peak Flow (aPF) was denoted as the largest exhaled flow rate from these 10 attempts within 0.67 L/s of another attempt. The same procedure was then performed to calculate the aEV and aPF of the stimulated breaths. Whether stimulation was applied during the first or second set of five MI-E cycles within each run was randomized at each session. It should be noted that aEV and aPF measure the volume and flow of the MI-E device combined with the participant's voluntary effort, and are therefore not directly comparable to FVC and PEF.

### Equipment

Baseline FVC and PEF measures were recorded using a spirometer (Microloop, CareFusion, San Diego, CA). MI-E was applied using a commercially available MI-E system (CoughAssist, Phillips Respironics, Amsterdam, Netherlands) via a mouthpiece. A nasal clip was used to prevent air leakage at the nose, with the participant instructed to form a seal at the mouth to prevent air leakage at this point. During MI-E the participant's respiratory function was measured using a spirometer (ML311, ADInstruments, Dunedin, New Zealand) that was connected to a respiratory flow head (MLT1000L, ADInstruments, Dunedin, New Zealand) and fitted in line with the tubing connecting the participant and the MI-E device. A pressure sensor (HDIM050GBZ8H5, First Sensor AG, Berlin, Germany) was connected to a filter, fitted in line with the tubing between the participant and the MI-E device as standard clinical practice, with the signal from this sensor used to detect the start of the insufflation and exsufflation periods.

The pressure sensor was interfaced with a laptop computer via a 14- bit USB data acquisition card (NI USB-6009, National Instruments Corp., Austin, TX, USA) and the spirometer used during MI-E was interfaced with this computer via a 16-bit data acquisition card (NI USB-6036E, National Instruments Corp., Austin, TX, USA). The spirometer used to measure baseline respiratory values was interfaced with the laptop computer using an RS232 connection. The pressure sensor signal was high pass filtered online to remove signal bias (1st order Butterworth, cut off frequency 0.04 Hz) and then differentiated. Data was recorded in the Simulink modelling environment (The Mathworks, Natick, MA, USA) using custom-made blocks to enable real time execution, with all data being recorded at a sample rate of 50 Hz. Data analysis was performed using MATLAB (version R2013a, The Mathworks, Natick, MA, USA).

### Stimulation system

**Figure 1:**
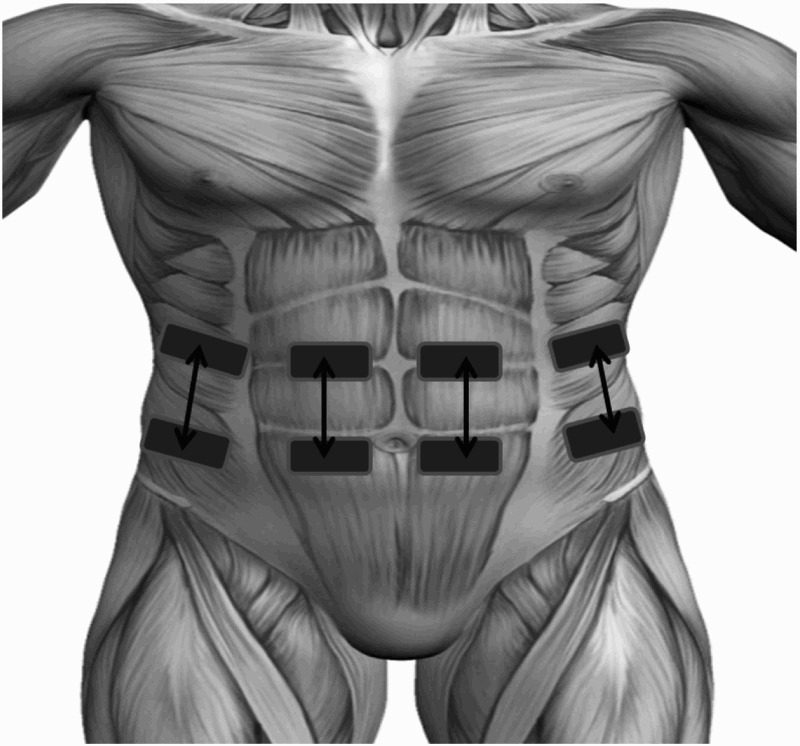
Schematic diagram of electrode placement showing four electrode pairs positioned to stimulate the motor points of the external oblique (outer electrodes) and rectus abdominis (inner electrodes) muscles on both sides of the body.

A programmable neuromuscular stimulator (Rehastim v1, Hasomed, Magdeburg, Germany) was used to stimulate the rectus abdominis and external oblique muscles bilaterally using four stimulation channels. Bi-phasic current controlled stimulation pulses (current 40 mA, pulsewidth 100 *µ*s) were applied at a frequency of 30 Hz for the whole exsufflation period (three seconds). Stimulation was applied via surface electrodes (33 mm×53 mm rectangular, PALS, Axelgaard, Fallbrook, CA, USA) placed over the motor points of the rectus abdominis and external oblique muscles on both sides of the body, as shown in Figure 1. Motor points were detected using a previously described technique.^13^

**Figure 2:**
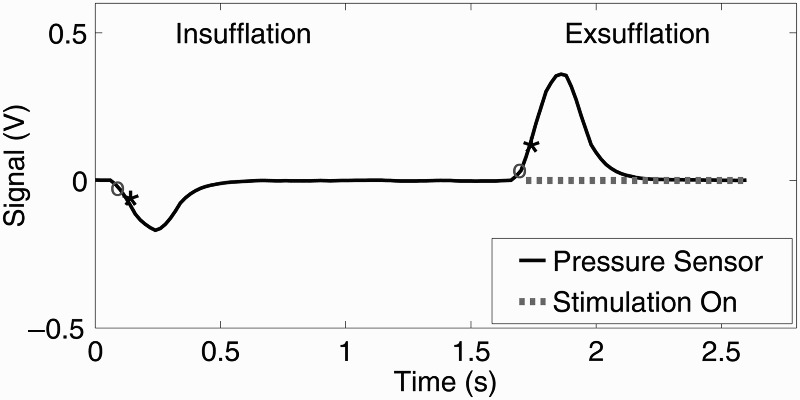
Pressure sensor signal recorded at assessment session six (A6), providing a graphical representation of the stimulation triggering algorithm. Stimulation was automatically applied at the start of exsufflation, identified as a sample greater than 0.05 V (shown by black ∗) proceeding a sample of less than 0.05 V (shown by grey o), when the previous zero crossing was an insufflation (detected using the opposite logic as shown at 0.1 s). The point where stimulation is applied during exsufflation is represented by a dotted grey line.

At the first two assessment sessions AFES was manually applied at the start of exsufflation by the researcher. At the remaining six assessment sessions AFES was automatically synchronized with MI-E using the signal from the pressure sensor, providing a total of 60 automatically stimulated breaths. AFES was automatically applied at the start of exsufflation, identified from the differentiated pressure sensor signal as a sample with a magnitude of greater than 0.05 V (0.05 V was used to avoid noise around the zero point) that proceeded a sample with a magnitude of less than 0.05 V, when the previous zero crossing was an insufflation (detected using the opposite logic). Additional logic was used to prevent AFES from being applied within two seconds of the previous stimulation burst ending. A graphical representation of this triggering algorithm applied to the pressure sensor signal, is shown in Figure 2. The total set up time at each session, consisting of donning electrodes and linking the pressure sensor with the cough assist system, was approximately five minutes.

### Analysis

A paired Student's *t*-test was used to test for a statistically significant difference (P < 0.05) between the absolute values of stimulated and unstimulated aEV and stimulated and unstimulated aPF across the eight assessment sessions.

The accuracy of the triggering algorithm was evaluated by comparing the time point at which stimulation was automatically applied using the signal from the pressure sensor with the respiratory flow recorded by the spirometer. If stimulation was applied within 0.2 s of the start of exsufflation, as detected by the spirometer, stimulation triggering was deemed successful. Analysis of triggering accuracy was performed on the 10 automatically stimulated breaths at each of the six assessment sessions where automatic stimulation was applied (A2 to A7), providing a total of 60 breaths. The percentage of these breaths that were correctly stimulated within 0.2 s of the start of exsufflation was calculated. A classification accuracy of >95%, which would result in only one of every 20 coughs being incorrectly stimulated, was deemed clinically suitable.

## Results

The participant's baseline respiratory function measured at the first (A0) and final (A7) assessment session is shown in Table 1.

**Table 1:** Baseline measures of Forced Vital Capacity (FVC) and Peak Expiratory Flow (PEF) recorded at the initial (A0) and final (A7) assessment session.

Assessment Session	FVC (L)		PEF (L/s)	
	Unstimulated	Stimulated	Unstimulated	Stimulated
**A0**	1.60	1.84	1.97	2.18
**A7**	1.99	2.28	2.00	2.39

AFES was successfully applied within 0.2 s of the start of exsufflation during 58 of the 60 (96.7%) recorded attempts. This classification performance was deemed clinically acceptable.

The absolute unstimulated aEV at the first (A0) and final (A7) assessment sessions was 1.12 L and 2.69 L, respectively. The absolute unstimulated aPF at the first (A0) and final (A7) assessment sessions was 6.22 L/s and 8.64 L/s, respectively.

**Figure 3:**
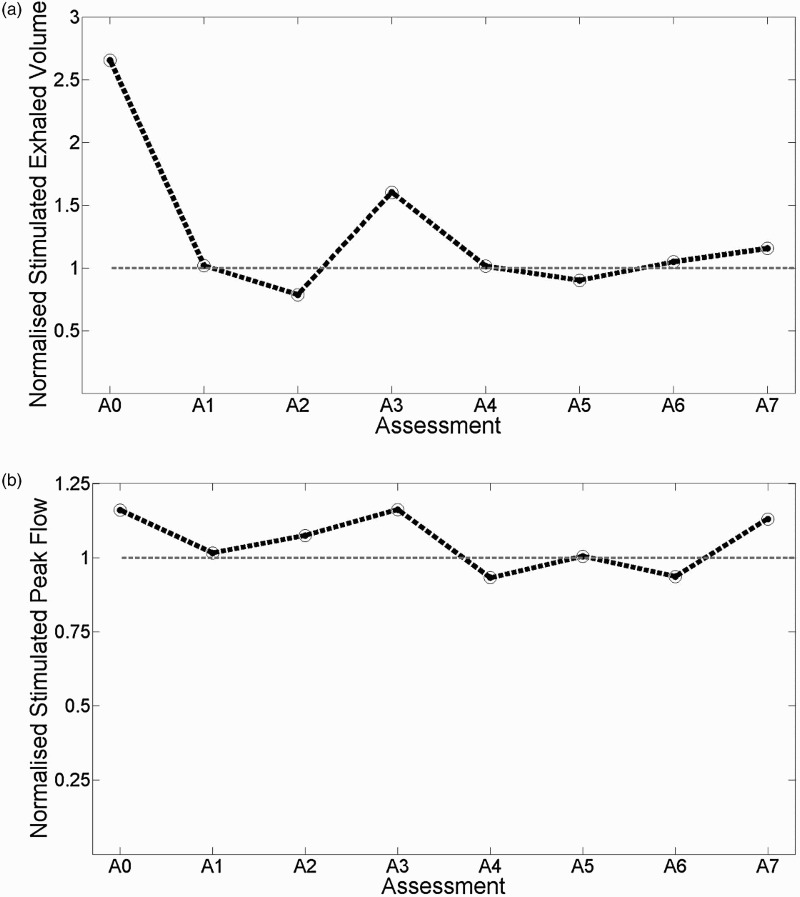
Stimulated assisted Exhaled Volume (aEV, plot (a)) and assisted Peak Flow (aPF, plot (b)) as a percentage of the unstimulated aEV and aPF at each assessment session. Unstimulated aEV and aPF is represented by a value of 100% (shown by grey dotted line). aEV and aPF were recorded during Mechanical Insufflation-exsufflation with one participant at eight fortnightly assessment sessions.

Figure 3 shows the participant's stimulated aEV and aPF as a percentage of the unstimulated aEV and aPF at each of the eight assessment sessions. The application of AFES achieved an acute increase in aEV and aPF at six of the eight (75%) assessment sessions (A0, A1, A3, A4, A6 and A7 for aEV;A0, A1, A2, A3, A5, and A7 for aPF). The difference between stimulated and unstimulated aEV (P = 0.64) and aPF (P = 0.44) was not found to be statistically significant. While this lack of statistical significance can largely be attributed to the small sample size (eight stimulated and unstimulated values collected from one participant), the finding that stimulated aEV and aPEF was greater than unstimulated aEV and aPF at the majority of assessment sessions has the potential to be clinically significant.

## Discussion

The aim of this study was to evaluate the feasibility of combining AFES with MI-E. The feasibility of the technique was demonstrated by the successful integration of AFES with MI-E at eight assessment sessions. The feasibility of this technique was further confirmed by the high (>95%) accuracy achieved when using the signal from a pressure sensor to automatically apply AFES during MI-E.

AFES assisted aEV and aPF were found to be greater than unstimulated aEV and aPF at 75% of assessment sessions. The aim of MI-E is to achieve secretion loosening by generating as high an aEV and aPF as possible. While the sample size in this study meant that statistical significance was not achieved, the increase in aEV and aPF observed when combining AFES with MI-E suggests that this combination may be more effective for secretion clearance than MI-E alone. For people with tetraplegia, more effective secretion clearance should reduce the risk of developing a respiratory complication, a leading cause of rehospitalisation for this patient group.^1^ Furthermore, more effective secretion clearance has the potential to benefit other patient groups with reduced respiratory function, such as those with COPD or neurodegenerative disorders. Therefore, the results in this study indicate that the effectiveness of combining AFES with MI-E warrants further investigation. Due to the non-invasive nature of AFES, positive results from such studies would suggest that the integration of AFES with MI-E should be considered for all future uses of this technology.

An AFES training protocol, which acts to strengthen the abdominal muscles through the repeated application of AFES, has been shown to lead to a longitudinal improvement in cough function.^14,15^ It is hypothesized that the implementation of such a training protocol may lead to longitudinal improvements in aEV and aPF recorded during MI-E. This could further improve secretion clearance, further reducing the chances of developing a respiratory complication. The absolute aEV, which represents the output of the MI-E device combined with the participant's voluntary effort, recorded at assessment session four (A4) was far greater than the aEV recorded at the other assessment sessions (approximately 6L at A4 versus less than 3L at other assessment sessions). As this value met the ATS/ERS standards, and as the stimulated and unstimulated values are similar, it is believed that this was not a measurement error. One possibility is that the MI-E device was on a different setting at this session compared to the other sessions. This abnormal result, combined with the natural recovery expected to be observed during sub-acute spinal cord injury, meant that a longitudinal comparison of respiratory function was outside the scope of this feasibility study.

One limitation of this study is that the MI-E pressures used, an insufflation pressure of 20 cmH_2_ *O* and an exsufflation pressure of −20 cmH_2_ *O*, are less than the pressures commonly recommended for clinical practice (40 cmH_2_ *O* and –40 cmH_2_ *O*).^3,6^ This pressure range was selected because the participant did not require acute clearance of secretions and it was believed that a pressure of 40 cmH_2_ *O* may be uncomfortable for prolonged testing. However, the lower pressures used in this study have the potential to increase patient comfort and may also reduce the risk of barotrauma, a known risk of MI-E.^16^ Furthermore, these pressures may enable AFES assisted MI-E to be used when clinical circumstances dictate that manually assisted coughing is not appropriate, as would likely be the case for patients with an unstable chest or abdominal injury. Therefore, while the pressures used in this study are lower than those recommended for clinical practice it is proposed that a range of pressures require to be investigated to fully ascertain the effectiveness of AFES combined with MI-E.

To fully evaluate the effectiveness of AFES combined with MI-E it would be desirable to also compare respiratory function during manually assisted coughing combined with MI-E. As the force required for a successful manually assisted cough is subjective, the same caregiver would be required to apply all manually assisted coughs in such a study. This was not possible in this study due to shift rotations. Due to the spirometer being placed in line with the participant and the MI-E device, the output of the MI-E device was measured along with the participant's voluntary effort. Therefore, aEV is reported as opposed to FVC. In future studies it would be beneficial to establish a method of measuring the user's FVC during MI-E. One possible solution is to apply a few cycles of MI-E and then measure an unassisted exsufflation after an assisted insufflation, a technique previously used by Sivasothy *et al.* to measure flow and volume after MI-E. However, further technological development is required to enable AFES to be applied automatically using this method. Finally, while this study demonstrates the feasibility of combining AFES with MI-E, as well as the novel use of a pressure sensor to automatically synchronize AFES with MI-E, results are reported from only one participant. A larger clinical trial evaluating respiratory function during AFES and manually assisted coughing with MI-E, and employing appropriate statistical testing to measure the significance of the treatment effect, is required to fully demonstrate the effectiveness of this technique.

## Conclusions

The results of this study show that AFES can feasibly be combined with MI-E. The acute increases in respiratory function observed at the majority of assessment sessions generate the hypothesis that AFES combined with MI-E may be more effective for secretion clearance than MI-E alone, which may reduce the likelihood of developing a respiratory complication.

**Trial Registration:** Clinicaltrials.gov NCT01800409
